# High-dose vitamin C: A promising anti-tumor agent, insight from mechanisms, clinical research, and challenges

**DOI:** 10.1016/j.gendis.2025.101742

**Published:** 2025-06-27

**Authors:** Hanzheng Zhao, Wentao Fu, Xiaobao Yang, Wenhui Zhang, Si Wu, Jingxin Ma, Tianzhen Zhang, Hongwei Yao, Zhongtao Zhang

**Affiliations:** a Department of General Surgery, Beijing Friendship Hospital, Capital Medical University, Beijing 100050, China; b National Clinical Research Center for Digestive Diseases, Beijing 100050, China; c Department of Hernia and Abdominal Wall Surgery, Beijing Chaoyang Hospital, Capital Medical University, Beijing 100020, China; d Department of Anesthesiology, Beijing Tongren Hospital, Capital Medical University, Beijing 100176, China; e Department of Clinical Laboratory, Beijing Friendship Hospital, Capital Medical University, Beijing 100050, China

**Keywords:** Ascorbic acid, Cancer therapy, Clinical trials, High-dose, Intravenous injection, Vitamin C

## Abstract

Vitamin C, also known as ascorbic acid, has sparked controversy since it first emerged as a potential anti-cancer agent. However, an increasing number of preclinical studies have demonstrated that high-dose vitamin C exhibits selective anti-tumor effects, including “pro-oxidative cytotoxicity”, “anti-cancer epigenetic regulation”, and “immune modulation”. Consequently, vitamin C has reemerged as a promising anti-cancer therapy in the form of high-dose administration. Advancements in pharmacokinetic research have facilitated the development of clinical trials. Early clinical studies across various cancer types have confirmed the safety of high-dose vitamin C administered via intravenous injection. Moreover, its use as an adjuvant therapy in combination with standard treatments, such as chemotherapy and radiotherapy, has shown promising therapeutic potential. However, there remains a lack of consensus regarding optimal dosage, administration methods, tumor specificity, and patient selection. These factors have contributed to the inconsistent outcomes observed in phase II clinical trials and have hindered the widespread conduct of phase III trials. Without robust clinical evidence, high-dose vitamin C, despite being a non-toxic and promising anti-cancer agent, risks being “shelved” once again. In this review, we provide a comprehensive overview of the anti-tumor mechanisms of high-dose vitamin C and a detailed analysis of preclinical and clinical studies investigating its role as an anti-cancer agent. Additionally, we explore emerging trends in high-dose vitamin C therapy for cancer treatment and offer recommendations for future research in this field.

## Introduction

Vitamin C, also known as ascorbic acid, is an essential nutrient that plays a crucial role in human physiology. As humans lack the key enzyme l-gulonolactone oxidase in the final step of vitamin C synthesis, they must obtain it from dietary sources to meet physiological needs.^1^^,^^2^ Numerous studies have demonstrated the organ-protective effects of vitamin C in the nervous, cardiovascular, gastrointestinal, coagulation, and immune systems.^3^ Consequently, vitamin C has been widely used in clinical settings for the treatment of various diseases, including ischemia-reperfusion injury,^4^ atherosclerosis,^5^ burns,^6^ sepsis and septic shock,^7^^,^^8^ and COVID-19.^9^

Since McCormick WJ first reported the potential anti-tumor effects of vitamin C in 1959,^10^ this nutrient has garnered significant interest as an antineoplastic agent, ushering in its first wave of attention. In the 1970s, Cameron and Pauling conducted matched-control studies showing that high-dose vitamin C (HDVC) supplementation could alleviate symptoms and prolong survival in advanced cancer patients.^11^^,^^12^ However, two subsequent rigorous prospective clinical trials by Creagan and Moertel failed to confirm the same outcome.^13^^,^^14^ The inconsistency in research results led to intense debates, and due to the double-blind prospective design of Creagan's study, the prevailing view at the time was that HDVC had no significant impact on cancer survival outcomes.

Recent advancements in the pharmacokinetics of vitamin C have provided new insights into these discrepancies. It is now understood that plasma concentrations of orally administered vitamin C are strictly controlled by gastrointestinal absorption, saturation of tissue transport proteins, and renal reabsorption and excretion. As a result, oral vitamin C can only achieve a maximum plasma concentration of 220 μmol/L.^15^^,^^16^ In contrast, intravenous administration bypasses these regulatory mechanisms, allowing plasma vitamin C concentrations to reach the millimolar range.^17^ Furthermore, preclinical studies have shown that “pharmacological concentrations” of vitamin C (20–30 mmol/L) have cytotoxic effects on cancer cells.^18^ These findings provide a potential theoretical explanation for the conflicting results in earlier clinical studies between Cameron's study (using extracorporeal plus oral administration) and Creagan's study (using oral administration only). As a result, intravenous HDVC as a potential cancer therapy has once again gained attention.

To date, multiple *in vitro* preclinical studies have demonstrated that pharmacological concentrations of vitamin C exhibit cytotoxicity against various tumor cell lines while having no significant impact on normal cells.^19^, ^20^, ^21^ Moreover, *in vivo* preclinical studies have shown that extracorporeal administration of 4 g/kg vitamin C can achieve pharmacological concentrations in mouse plasma and significantly inhibit the occurrence and development of ovarian, pancreatic, and colorectal cancers.^22^, ^23^, ^24^ In the clinical setting, pharmacological concentrations of intravenous vitamin C have been proven to be safe and well-tolerated,^25^ prompting the initiation of subsequent clinical trials. However, clinical trials investigating the effects of vitamin C monotherapy for tumor control and survival have yielded conflicting results and shown limited therapeutic efficacy.^26^, ^27^, ^28^, ^29^ Furthermore, since the anti-tumor effects of vitamin C remain uncertain, conducting such studies in patients eligible for standard treatments raises ethical concerns. Given these considerations, future research on HDVC should prioritize its role as a supplement to standard therapies.

Currently, multiple preclinical studies have demonstrated the potential benefits of combining HDVC with existing anti-tumor treatments such as chemotherapy and radiotherapy, enhancing their therapeutic effects while reducing side effects.^30^, ^31^, ^32^ Clinically, strong evidence suggests that HDVC can reduce the side effects of chemoradiotherapy, such as fatigue and gastrointestinal reactions, and improve quality of life.^33^, ^34^, ^35^ However, the outcomes regarding treatment efficacy remain controversial, which may be related to differences in study designs (dosage, frequency, sequence, and patient selection). Therefore, despite HDVC showing promise as an anti-tumor agent, several critical issues remain to be addressed.

In this review, we first summarize the molecular mechanisms underlying the anti-tumor effects of HDVC. Then, we provide an overview of the latest promising preclinical and clinical studies on the use of HDVC. Finally, we discuss the emerging trends of HDVC in the field of cancer treatment. We attempt to answer the following questions: i) What are the potential mechanisms underlying the effects observed when HDVC is used for anti-tumor treatments? ii) How to identify the potential beneficiary patients for HDVC treatment and determine the optimal dosage, concentration, and sequence? iii) What is the future direction of research in this field? We also emphasize the limitations of current clinical research and highlight the necessity of conducting more rigorous trials to establish HDVC's therapeutic potential in oncology.

## Anti-tumor mechanisms

**Figure 1:**
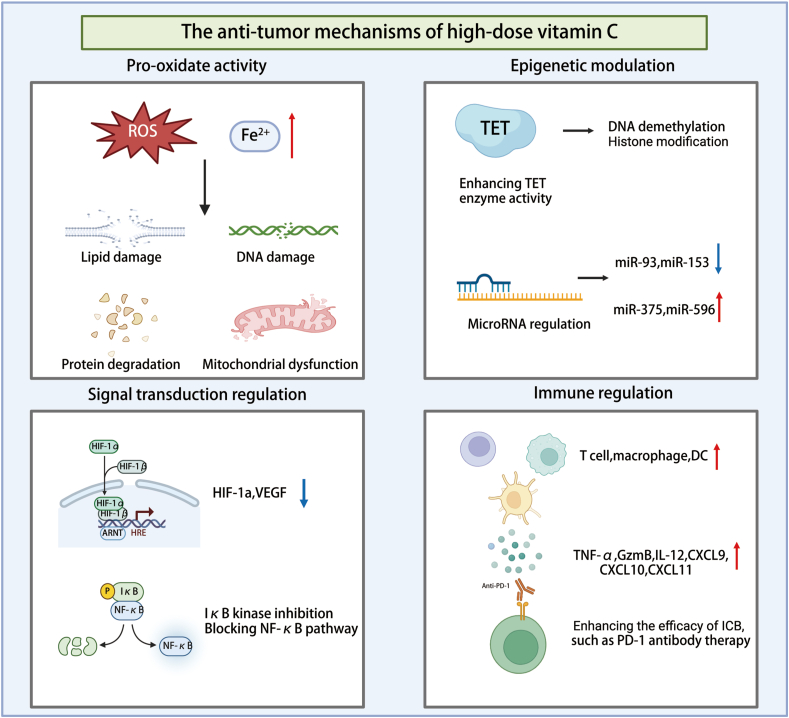
The anti-tumor mechanisms of high-dose vitamin C.

Studies have revealed that its anti-cancer effects are mediated by regulating oxidative stress, epigenetic modifications, signaling pathways, and immune responses (Fig. 1). The following sections will provide a detailed exploration of these mechanisms to elucidate the potential applications of vitamin C in cancer treatment.

### Pro-oxidant activity

**Figure 2:**
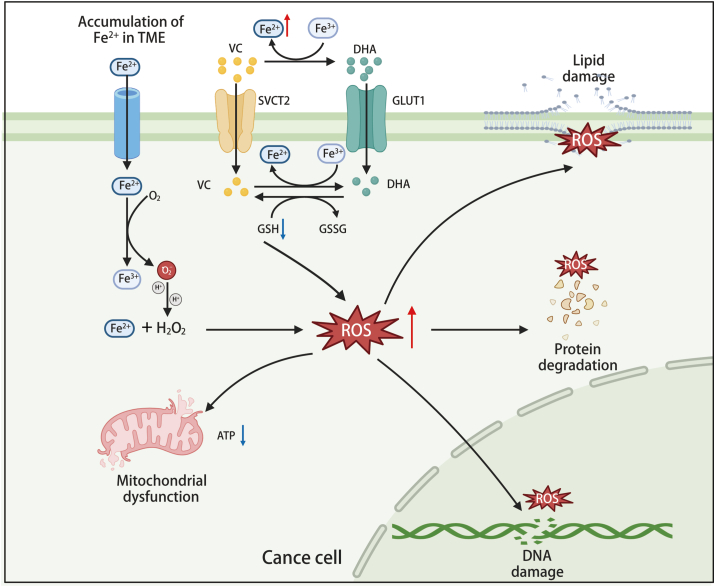
Elevated intracellular oxidative stress induced by high-dose vitamin C disrupts tumor cell redox balance and kills tumor cells.

Vitamin C exhibits distinct antioxidant and pro-oxidant properties depending on its concentration and route of administration.^36^ At low concentrations, vitamin C primarily acts as an antioxidant. However, at high concentrations, it displays pro-oxidant characteristics, directly inducing oxidative stress in cells, particularly within the tumor microenvironment (TME), where it exerts anti-tumor effects.^37^^,^^38^ HDVC induces the production of reactive oxygen species (ROS), which exerts a cytotoxic effect on tumor cells. The pro-oxidant effects of HDVC are mechanistically centered on two synergistic axes, namely, iron-dependent ROS generation and increased vitamin C uptake via glucose transporter 1 (GLUT1)/sodium-dependent vitamin C transporter 2 (SVCT2)-mediated transport, with the latter enabling tumor-selective accumulation of ascorbate. The underlying mechanisms are detailed in Figure 2.

#### Iron-dependent ROS generation

The selective toxicity of vitamin C in tumors is fundamentally linked to their unique iron metabolism. Tumor cells exhibit a significantly higher dependence on iron compared with normal cells, with an accumulation of labile iron playing a pivotal role in tumor initiation, progression, drug resistance, and immune evasion.^39^ For instance, the labile iron levels in breast cancer cells are approximately twice those in normal breast epithelial cells.^40^ This iron dependency provides a potential target for vitamin C's pro-oxidant activity against tumor cells. High concentrations of vitamin C can react with iron ions to generate hydrogen peroxide (H_2_O_2_), which, through the Fenton reaction, produces hydroxyl radicals (·OH).^20^^,^^41^ These highly reactive radicals not only induce DNA damage but also disrupt cell membrane integrity, ultimately leading to tumor cell death.^38^

At pharmacologic concentrations, typically achieved via intravenous administration, the pro-oxidant effects of vitamin C are particularly pronounced.^22^^,^^42^ Vitamin C promotes the redox cycling of Fe^2+^ and Fe^3+^, leading to sustained production of H_2_O_2_ and hydroxyl radicals. This mechanism is particularly effective in cancers with high labile iron levels, such as multiple myeloma.^43^ Additionally, extracellularly generated H_2_O_2_ can diffuse into tumor cells, further exacerbating intracellular oxidative stress. This coordinated oxidative microenvironment selectively damages tumor cells while sparing normal cells. The intrinsic defense mechanism of cancer cells against oxidative stress induced by ascorbic acid is predominantly catalyzed by catalase. Consequently, inhibition of catalase renders these cells more susceptible to oxidative stress.^44^

#### Increased vitamin C uptake via GLUT1 or SVCT

Beyond iron metabolism, the metabolic reprogramming of cancer cells creates a unique vulnerability exploited by vitamin C. The Warburg effect—a hallmark of cancer metabolism characterized by increased glycolysis—directly facilitates vitamin C uptake in tumors. Tumor cells, particularly those with KRAS or BRAF mutations, rely heavily on glycolysis for energy production, leading to overexpression of GLUT1 and SVCT2. This overexpression not only enhances glucose uptake but also increases intracellular levels of vitamin C or its oxidized form, dehydroascorbic acid (DHA).^21^^,^^45^ In these highly glycolytic cells, DHA competes against glucose for GLUT1-mediated uptake. Once inside the cell, DHA is rapidly reduced to ascorbate, depleting NADPH reserves and generating oxidative stress.^46^ This leads to intracellular glutathione depletion and ROS accumulation, which oxidizes and inhibits GAPDH, a key glycolytic enzyme. The inhibition of GAPDH disrupts glycolysis, causing an energy crisis and cell death, particularly in KRAS and BRAF mutant cells. Additionally, the increase in ROS can activate poly(ADP-ribose) polymerase (PARP), further depleting NAD^+^, which exacerbates cellular oxidative stress. Importantly, HDVC selectively exploits this metabolic vulnerability. Unlike normal cells that maintain NAD^+^ and glutathione homeostasis, KRAS and BRAF mutant cells cannot cope with the oxidative stress and metabolic collapse induced by vitamin C. Studies have shown that HDVC impairs tumor growth in KRAS and BRAF mutant colorectal cancer models without affecting wild-type cells.^47^

In summary, the therapeutic potential of vitamin C in cancer treatment lies in its ability to exploit the pro-oxidant mechanism targeting tumor cell metabolic vulnerabilities. By interacting with the surplus labile iron in tumor cells, vitamin C generates extracellular H_2_O_2_, which is further converted into cytotoxic hydroxyl radicals. Moreover, enhanced uptake of glucose and DHA by tumor cells provides a metabolic advantage for vitamin C's selective cytotoxicity. These characteristics position vitamin C as a promising strategy for personalized cancer therapy, particularly for tumors with high expression of GLUT1 and SVCT2.^48^^,^^49^ Future studies should aim at optimizing its efficacy and safety through combination with other therapeutic modalities, paving the way for novel cancer treatment paradigms.

### Epigenetics: regulating gene expression

Ten-eleven translocation enzymes (TETs) are α-ketoglutarate (α-KG)- and Fe^2+^-dependent dioxygenases that play a pivotal role in DNA demethylation. Their activity is closely associated with the development of various diseases, including hematological malignancies.^50^ Studies have shown that vitamin C enhances TET enzyme activity, promoting DNA demethylation and reversing tumor-associated DNA hypermethylation patterns.^51^ Specifically, TETs oxidize 5-methylcytosine (5mC) to 5-hydroxymethylcytosine (5hmC), restoring expression of hypermethylation-silenced tumor suppressor genes such as SMAD1.^52^ In acute myeloid leukemia, TET2 mutations or functional loss result in global DNA methylation abnormalities. Vitamin C corrects these anomalies by activating residual TET proteins, facilitating myeloid cell differentiation, and ultimately inhibiting the proliferation of leukemia cells.^53^ This effect extends beyond hematological malignancies. In solid tumors such as melanoma^54^ and bladder cancer,^55^ vitamin C significantly reduces tumor malignancy by elevating 5hmC levels.

Moreover, vitamin C indirectly regulates miRNA expression by altering DNA methylation and histone modifications, thereby influencing tumor behavior.^56^ For instance, vitamin C suppresses oncogenic miRNAs like miR-93 and miR-153 while up-regulating tumor-suppressive miRNAs such as miR-375 and miR-596.^57^^,^^58^ These changes have profound impacts on tumor cell proliferation and invasion and are strongly correlated with patient survival.

Interestingly, vitamin C also modulates the immune microenvironment. For example, in pancreatic cancer cells, it down-regulates the expression of histone acetyltransferase 1 (HAT1),^59^ reducing the levels of the immune checkpoint molecule programmed cell death ligand 1 (PD-L1) and thereby inhibiting tumor immune evasion. However, whether TET activation consistently correlates with PD-L1 expression levels remains unclear. In lymphoma cells, while vitamin C enhances TET activity, it does not significantly alter PD-L1 levels.^60^

### Signal transduction regulation

#### HIF-1 signaling pathway

Hypoxia-inducible factor 1 (HIF-1) is a key transcription factor in the TME, responding to hypoxic conditions. Composed of HIF-1α and HIF-1β subunits, HIF-1α stabilizes under hypoxia by inhibiting prolyl hydroxylase activity, activating genes such as vascular endothelial growth factor (VEGF), GLUT1, and matrix metalloproteinase (MMP) that promote tumor proliferation, angiogenesis, and invasion.^61^

As a cofactor for HIF hydroxylases, vitamin C enhances their activity, promoting HIF-1α degradation and reducing its protein levels, thereby inhibiting HIF-1-mediated pro-tumorigenic effects.^62^^,^^63^ This action has been validated in various cancers, including melanoma,^64^ colorectal cancer,^65^ pancreatic adenocarcinoma,^66^ and thyroid cancer.^67^ Administration of vitamin C has been demonstrated to exert a dose-dependent inhibitory effect on the expression of HIF-1α and its downstream genes, including GLUT1 and VEGF.^68^ This has been shown to result in suppression of angiogenesis and tumor progression.

Clinical studies have shown an inverse correlation between vitamin C levels in tumor tissues and HIF-1α activity.^69^ In renal cell carcinoma with VHL deficiency, vitamin C reduces GLUT1 expression and increases ROS levels, inducing cell death and providing new avenues for targeted therapy.^70^

#### NF-κB signaling pathway

Nuclear factor kappa-B (NF-κB) is a critical transcription factor that plays a pivotal role in regulating the expression of genes associated with inflammation and tumor development.^71^ Abnormal activation of this factor is strongly associated with tumor formation. It enhances the survival and spread of cancerous cells by increasing expression of anti-apoptotic proteins, inflammatory cytokines, such as interleukin-6 (IL-6) and tumor necrosis factor-alpha (TNF-α), and molecules that promote metastasis.^72^

Vitamin C inhibits NF-κB activity through multiple mechanisms, demonstrating its anti-inflammatory and anti-tumor effects.^73^ On one hand, vitamin C can block the degradation and phosphorylation of inhibitor of nuclear factor kappa-B kinase subunit alpha (IκBα) mediated by interleukin-1 (IL-1) and TNF, thereby inhibiting activation of NF-κB.^74^ Studies have found that vitamin C inhibits the activation of NF-κB and the degradation of IκBα by activating the p38 pathway, thereby suppressing the inflammatory response and related pathological processes within cells.^75^ On the other hand, vitamin C enhances TET enzyme activity, modulating DNA demethylation and indirectly down-regulating NF-κB target gene expression.^76^ This dual regulatory mechanism not only mitigates tumor-associated inflammation but also increases tumor sensitivity to chemotherapy and radiotherapy.

Studies have shown that vitamin C can produce ROS within cells, which in turn inhibit the activity of inhibitor of nuclear factor kappa-B kinase (IKK), preventing the phosphorylation and degradation of IκB. This ultimately reduces NF-κB-mediated inflammatory responses.^77^

### Immune modulation: enhancing anti-tumor responses

The role of vitamin C in immune function regulation is increasingly being recognized, particularly in the field of cancer immunotherapy. It has demonstrated significant potential in enhancing the anti-tumor capabilities of immune cells through various mechanisms.^78^

Firstly, vitamin C helps maintain high levels of ascorbic acid within immune cells, which is critical for bolstering both innate and adaptive immune responses.^79^ Its role as an antioxidant is well-established, but it also acts as a vital cofactor for iron- or copper-containing oxygenases, profoundly affecting immune cell metabolism and function. For instance, vitamin C can enhance signal transducer and activator of transcription 3 (STAT3) binding to the regulatory region of the PR domain zinc finger protein 1 (Prdm1) gene via DNA demethylation, thereby promoting the differentiation of B cells into plasma cells.^80^ Additionally, during dendritic cell maturation, vitamin C pretreatment significantly demethylates NF-κB/p65 binding sites, up-regulating expression of antigen presentation-related genes and promoting the release of TNF-β, thereby enhancing immune responses.^76^

Within TME, vitamin C enhances the anti-tumor activity of immune cells through various mechanisms. It promotes the infiltration of CD4^+^ T cells, CD8^+^ T cells, and macrophages, increasing the production of granzyme B and interleukin-12 (IL-12), which significantly enhances immune cell-mediated anti-tumor activity.^81^ Notably, vitamin C demonstrates a synergistic effect with immune checkpoint inhibitors (such as programmed death-1 (PD-1) and cytotoxic T-lymphocyte-associated protein 4 (CTLA-4) antibodies).^60^^,^^82^ It also up-regulates chemokines like C-X-C motif chemokine ligand 9/10/11 (CXCL9/10/11), attracting more tumor-infiltrating lymphocytes into the tumor tissue, further boosting anti-tumor immune responses.^83^

The regulatory effects of vitamin C on specific immune cells are particularly noteworthy. For example, physiological doses of vitamin C significantly promote the proliferation and cytokine secretion of γδ T cells while inhibiting their early apoptosis, thereby enhancing their tumor-killing activity.^84^ Furthermore, vitamin C reduces ROS levels in γδ T cells, increasing their metabolic vigor and replicative capacity. For natural killer cells, vitamin C supplementation significantly increases their numbers and maintains their functional and phenotypic stability, further augmenting their cancer-killing capabilities.^85^ In addition, it has been demonstrated that vitamin C can mitigate the inflammatory response and impede the inflammatory state and angiogenesis of TME to a certain extent. This may potentially influence the progression of tumor growth and metastasis.^86^^,^^87^

Vitamin C also influences immune cell function through epigenetic regulation. It restores the activity of TET enzymes in regulatory T cells, stabilizing forkhead box P3 (Foxp3) expression, which is crucial for regulating immune cell polarization, differentiation, and adaptive immune functions.^88^ This highlights its broad involvement in the immune system, as it not only promotes maturation and activation of immune cells by regulating metabolism and gene expression but also optimizes immune responses through epigenetic mechanisms, particularly in inflammation and TME.^89^

In summary, vitamin C influences the immune system in multifaceted ways, including enhancing anti-tumor activities of natural killer cells and γδ T cells, promoting differentiation of dendritic cells and plasma cells, and improving regulatory T cell functionality through epigenetic regulation. Its potential as an immune modulator provides novel insights and applications for cancer immunotherapy. Furthermore, its synergistic effect with immune checkpoint inhibitors underscores its value in enhancing anti-tumor responses, offering a strong foundation for further research and clinical translation.

## Preclinical studies on HDVC

### Single-agent therapy

HDVC has shown selective cytotoxicity against tumor cells *in vitro*, with its anti-tumor efficacy strongly associated with its concentration. Different concentrations of vitamin C elicit distinct biological responses. Studies have indicated that when the plasma concentration of vitamin C exceeds 1 mmol/L, it can directly induce tumor cell necrosis.^90^ The tolerance of normal cells to high concentrations of vitamin C is primarily attributed to differences between normal and cancer cells in vitamin C metabolism and glucose transporter expression. These differences make cancer cells more vulnerable to hydrogen peroxide-induced damage and antioxidant depletion, leading to DNA damage, energy exhaustion, and ultimately cell death. Research has revealed that vitamin C can function as an inhibitor of the Warburg effect, effectively slowing down the proliferation rate of cancer cells, neutralizing the carcinogenic effects induced by glucose, while causing relatively minimal damage to healthy cells.^91^

HDVC exerts its effects by disrupting iron metabolism and promoting oxidative stress, thereby generating high levels of ROS that induce oxidative stress within cells. ROS can directly damage DNA and interfere with DNA repair pathways, further impairing tumor cell survival and selectively inducing tumor cell death.^92^ This effect is particularly evident in certain tumor types, such as osteosarcoma^93^ and non-small cell lung cancer,^94^ which are highly sensitive to ROS, as well as tumors harboring specific genetic mutations such as KRAS or BRAF mutations.^47^^,^^95^

Recent studies have also highlighted the potential of HDVC to modulate the TME and enhance immune responses. For instance, HDVC has been shown to suppress PD-L1 expression in breast cancer cell lines, thereby reducing tumor immune evasion. Additionally, it can enhance the activity and cytotoxicity of CD8^+^ T cells.^96^ Moreover, HDVC activates TET2 and regulates the cyclic GMP‒AMP synthase (cGAS)/stimulator of interferon genes (STING) signaling pathway, facilitating tumor vascular normalization, improving the TME, and further boosting the efficacy of immunotherapy.^97^ Interestingly, vitamin C can enhance tumor immune responses by promoting expression of antigen presentation genes and interferon-gamma (IFN-γ) signaling through activation of TET2.^98^

In animal models, intraperitoneal or intravenous administration of HDVC (1–4 g/kg body weight, particularly at 4 g/kg body weight) has been demonstrated to effectively inhibit tumor growth across various cancer types.^99^, ^100^, ^101^ However, while HDVC exhibits selective cytotoxicity against tumor cells, its long-term safety in normal tissues remains to be fully evaluated, particularly regarding potential systemic oxidative stress induced by high concentrations.

In summary, these preclinical studies have validated the anti-tumor potential and mechanisms of HDVC, especially its ability to selectively kill tumor cells and modulate immune responses, which provides a solid foundation for further research.

### Combination therapy

Beyond its stand-alone therapeutic effects, its synergistic impact when combined with radiotherapy, chemotherapy, targeted therapy, and immunotherapy further stresses its promising role in cancer treatment. This review systematically elaborates on the recent progress in understanding the role of HDVC across various therapeutic strategies.

The role of HDVC in radiotherapy is multifaceted, particularly in alleviating radiation-induced damage. Studies have shown that it effectively inhibits the transformation of fibroblasts into myofibroblasts, thereby mitigating radiation-induced pulmonary fibrosis. In a mouse model of lung cancer, a combination of HDVC with radiotherapy significantly enhanced tumor growth inhibition, demonstrating its potential as a radiosensitizer. These findings suggest that HDVC not only reduces radiotherapy-associated side effects but also enhances its anti-tumor efficacy.^102^

In the realm of chemotherapy, HDVC exerts notable synergistic effects by modulating tumor cell metabolic pathways and impairing their antioxidant defense systems. Research has shown that HDVC decreases intracellular glutathione levels, thereby enhancing the cytotoxicity of chemotherapeutic agents such as carboplatin,^103^ cisplatin,^104^ and gemcitabine.^105^ Additionally, it regulates the citrate metabolism pathway in tumor cells, further sensitizing them to chemotherapeutic agents.^106^ For instance, in uterine serous carcinoma cells, a combination of HDVC and carboplatin significantly increased cellular stress responses and DNA damage, thereby inhibiting tumor invasion. This metabolic regulatory mechanism provides novel insights for improving chemotherapy efficacy.^107^

A combination of HDVC with targeted therapies has shown considerable promise in cancer treatment. By generating excessive H_2_O_2_ and inducing oxidative stress, HDVC selectively promotes cancer cell apoptosis while exhibiting low toxicity to normal cells. This forms a foundation for its synergistic effects with targeted agents. Targeted drugs inhibit key signaling pathways, impairing cancer cells' metabolic adaptability and antioxidant defenses. When combined with HDVC, this approach not only suppresses tumor proliferation but also reduces resistance development. For example, in colorectal cancer and non-small cell lung cancer, HDVC combined with epidermal growth factor receptor (EGFR) and PARP inhibitors demonstrated superior therapeutic outcomes.^31^^,^^108^ Mechanistic studies revealed that HDVC disrupts the redox balance in cancer cells, leading to cell cycle arrest, DNA damage accumulation, and metabolic disruption, thereby significantly enhancing therapeutic efficacy.

A combination of HDVC with immunotherapy offers a new avenue in cancer treatment. Research has shown that HDVC modulates the TME by inhibiting the activity of immunosuppressive cells, such as regulatory T cells and myeloid-derived suppressor cells, while enhancing the function of effector T cells and natural killer cells.^78^ HDVC combined with immune checkpoint inhibitors significantly enhanced tumor growth suppression and improved long-term survival rates. This combination therapy increased the proportion of tumor-infiltrating CD8^+^ T cells and down-regulated immunosuppressive factors.^60^ Furthermore, it reduced the occurrence of resistance to immunotherapy, supporting its multi-level synergistic mechanisms.

HDVC also exhibits significant effects when combined with other therapeutic modalities. For instance, arsenic trioxide plays a critical role in acute promyelocytic leukemia treatment, and HDVC enhances tumor cell sensitivity to arsenic trioxide by inhibiting key enzymes in glycolysis while reducing its toxic side effects.^109^ Furthermore, a combination of HDVC and auranofin demonstrated remarkable anti-tumor efficacy in a spectrum of leukemia cell lines, effectively impeding tumor cell proliferation and stimulating their apoptosis.^110^

In conclusion, as a multifunctional adjuvant therapeutic agent, HDVC exhibits significant synergistic effects with radiotherapy, chemotherapy, targeted therapy, and immunotherapy through mechanisms involving metabolic regulation, antioxidant defense attenuation, and enhanced treatment sensitivity. Its diverse mechanisms and broad applicability in preclinical studies provide a strong foundation for future clinical practice. These promising preclinical studies discussed herein are presented in Table 1.

**Table 1:** Preclinical studies on the inhibitory effects of vitamin C on tumor progression.

Cancer type	Cell	Dose of vitamin C	Results	Reference
Thyroid cancer	8305C, BCPAP, 8505C, FTC133 and TPC-1 cell lines	*In vitro*: 0.5, 1, 2, and 4 mM *In vivo*: 4 g/kg	Inhibiting thyroid cancer cell proliferation and promoting apoptosis; eliminating thyroid cancer cells by targeting the MAPK/ERK and PI3K/AKT pathways through a reactive oxygen species-dependent mechanism	38
Multiple myeloma	ARP1 and OCI-MY5 cell lines	*In vitro*: 2 and 4 mM *In vivo*: 4 mg/kg	Destroying tumor cells by generating reactive oxygen species in the presence of iron; selective killing of CD138^+^ multiple myeloma cells from multiple myeloma and smoldering multiple myeloma	43
Cholangiocarcinoma	CCLP1, TFK1, RBE, HCCC9810, and HuCCT cell lines	*In vitro*: 0.3, 0.5 and 2 mM *In vivo*: 4 g/kg	Inducing cytotoxicity in cholangiocarcinoma cells through the generation of intracellular reactive oxygen species, which results in DNA damage, ATP exhaustion, and suppression of the mTOR signaling pathway	45
Bladder cancer	T24, 5637, UMUC-3, and J82 cell lines	*In vitro*: 0.1, 0.5, and 1 mM *In vivo*: 2 g/kg	Increasing the content of 5hmC reduces the malignant phenotypes of bladder cancer	55
Melanoma	WM1366 and WM9 cell lines	*In vitro*: 10, 25, 50, 100, 250, and 500 μM	Inhibiting the progression of melanoma by reducing the expression of HIF-1α	64
Lymphoma	A20, SU-DHL-6, OCI-Ly1, OCI-Ly7, and OCI-Ly3 cell lines	*In vitro*: 1 mM	Promoting tumor infiltration by CD8^+^ T cells and macrophages and enhancing granzyme B levels in cytotoxic T cells and natural killer cells; increasing interleukin-12 release from antigen-presenting cells	60
Colorectal cancer	DiFi, IRCC-10A, CCK81, and C75 cell lines	*In vitro*: 1 mM *In vivo*: 4 g/kg	Combining with cetuximab to inhibit the emergence of secondary resistance to EGFR blockade in RAS/BRAF wild-type colorectal cancer patients	158
Melanoma, colorectal cancer	B16-OVA and MC38 cell lines	*In vivo*: 4 g/kg	Activating TET activity to increase the infiltration of tumor-infiltrating lymphocytes; enhancing the efficacy of anti-PD-L1 therapy	83

## Clinical studies on HDVC

The success of preclinical studies has paved the way for the initiation of clinical trials. Currently, several phase I and II clinical studies have explored the therapeutic effects of pharmacological concentrations of vitamin C in combination with standard anti-tumor regimens (Table 2).

**Table 2:** Clinical studies using HDVC as a combination therapy with standard anti-tumor regimens.

Combination treatment	Cancer type	Phase allocation (number of enrolled patients)	Vitamin C dose and administration route	Result	Conclusion	Reference
Chemotherapy (gemcitabine and nab-paclitaxel)	Pancreatic cancer	Phase-II randomized trial (34 patients)	Intravenous, 75 g, 3 times weekly, 4-week cycles	Safe and well tolerated; average plasma ascorbate concentration at the baseline and the end of every 2 cycles was 16 ± 3 mM in arm 2; favorable PFS and OS compared with gemcitabine and nab-paclitaxel alone, without a negative impact on the quality of life or an increase in the frequency or severity of adverse events	This randomized, actively controlled trial provides key data regarding effect size to design a phase III trial	83
Chemotherapy (gemcitabine)	Pancreatic cancer	Phase-I single-arm trial (9 patients)	Intravenous, 15–125 g, 4 week cycles (patients completing at least 2 cycles), 2 times weekly, dose-limiting criteria were not met	Safe and well tolerated; 50–125 g twice weekly produced plasma levels of at least 350 mg/dL (20 mM); favorable PFS and OS compared with historical controls	A Phase II study is warranted	113
Chemotherapy (gemcitabine and erlotinib)	Pancreatic cancer	Phase-I single-arm trial (14 patients)	Intravenous, dose escalation design, 50 g, 75 g, 100 g/infusion; 8-week cycles, 3 times per week	Safe and well tolerated; patients receiving 100 g/infusion whose plasma ascorbate level was between 25.3 and 31.9 mmol/L; favorable PFS and OS compared with historical controls	Outcomes are consistent with preclinical experiments; a phase II study is warranted	120
Chemotherapy (carboplatin and paclitaxel)	Non-small cell lung cancer	Phase-II single-arm trial (38 patients)	Intravenous, 75 g, 2 times weekly, every 3 weeks for 4 cycles	Safe and well tolerated; average post-infusion plasma ascorbate concentration was 17.6 mM; significantly improved tumor response rate compared with the historical control; favorable PFS and OS compared with the historical controls; participants with longer PFS demonstrated a higher fold increase in circulating activated effector CD8 T cells	The introduction of HDVC not only improved the treatment effect but also appeared to alter the host immune response; further investigation as a potential adjuvant to immunotherapy is warranted	122
Chemotherapy (carboplatin and paclitaxel)	Ovarian cancer	Phase-I/IIa randomized trial (27 patients)	Intravenous, dose escalation design, 75–100 g, 22 times weekly, for 12 months	Safe and well tolerated; plasma ascorbate concentration of 20–23 mM; decreased chemotherapy toxicities; favorable PFS and a trend to improve OS compared with carboplatin and paclitaxel only	Larger clinical trials are warranted	30
Chemoradiotherapy	Glioblastoma	Phase-I single-arm trial (11 patients) Phase-II single-arm trial (55 patients)	Intravenous, 87.5 g, 3 times weekly during radiotherapy, 2 times weekly during chemotherapy	Safe and well tolerated; 87.5 g dose consistently achieved plasma ascorbate concentrations of ≥20 mM in both the radiation therapy and chemotherapy phases; favorable PFS and OS compared with historical controls	Larger phase III clinical trials are warranted; T2∗-based MRI assessment of tumor iron content is a prognostic biomarker for glioblastoma clinical outcomes	115,121
Targeted therapy (bevacizumab)	Colorectal cancer	Phase-III randomized trial (442 patients)	Intravenous, 1.5 g/kg/d, maximum of 12 cycles or until disease progression	Safe and well tolerated; in the whole cohort, PFS and OS were not favorable compared with the control group, but in subgroup analysis, patients with RAS mutation in the HDVC group had longer PFS compared with the control group and tended to have a longer OS (but failed to achieve statistical significance)	This phase III trial suggested that future clinical trials should take patient stratification into account	125
Immunotherapy (pembrolizumab)	Metastatic leiomyosarcoma	Case report (2 patients)	Intravenous, 25 g per week	Safe and well tolerated; patients show persistent partial tumor remission	This HDVC in combination with immunotherapy study suggested that HDVC has the potential to improve immunotherapy effect; further trials are warranted	123
Non-pharmaceutical therapy (modulated electro-hyperthermia)	Non-small cell lung cancer	Phase-II randomized trial (97 patients)	Intravenous, 1.5 g/kg/d, 3 times a week for 25 treatments	Safe and well tolerated; significantly improved quality of life and favorable PFS and OS compared with the best supportive care group	Larger phase III clinical trials are warranted	127

Note: PFS, progression-free survival; OS, overall survival.

Safety is a critical prerequisite for conducting clinical trials. Consistent with preclinical studies, almost all studies combining HDVC with chemoradiotherapy have reported no potential toxicity associated with HDVC. Moreover, multiple studies have shown that HDVC can reduce the side effects caused by chemotherapy or radiotherapy.^30^^,^^111^, ^112^, ^113^, ^114^, ^115^ Additionally, research has indicated that HDVC does not interfere with the pharmacokinetics of chemotherapeutic drugs.^116^ As vitamin C is metabolized to oxalate and excreted in the urine, HDVC has raised concerns about a possible increased risk of urolithiasis.^117^ However, studies on both monotherapy and polytherapy have demonstrated satisfactory HDVC metabolism in cancer patients with normal renal function, with less than 0.5% of ascorbic acid being recovered as urinary oxalate, indicating a low risk of urolithiasis.^118^^,^^119^ These findings support the safety of HDVC in combination with standard treatment in clinical trials as presented in Table 2.

### Combination with chemotherapy and radiation therapy

The majority of HDVC combination treatment studies have been conducted alongside chemotherapy and/or radiation therapy, involving various tumor types. Consistent with the positive findings from preclinical studies, several phase I/II studies have reported prolonged survival rates and trends of disease stabilization or remission in patients receiving combination therapies. A phase I single-arm study found that HDVC combined with chemotherapy (gemcitabine) resulted in primary tumor shrinkage in 8 out of 9 patients, with specific shrinkage observed in subjects receiving the highest dose of ascorbic acid. This degree of shrinkage in stage IV pancreatic cancer is not typically found with stand-alone gemcitabine or gemcitabine combined with erlotinib treatment in clinical settings.^120^ In another phase I single-arm study initiated by Welsh et al^113^ in pancreatic cancer patients, ascorbic acid combined with standard chemotherapy (gemcitabine) showed significantly prolonged progression-free survival (PFS) and overall survival (OS) compared with historical controls. A phase I single-arm study conducted by Buatti et al^115^ in glioblastoma patients demonstrated that HDVC combined with chemoradiotherapy (radiotherapy plus temozolomide) resulted in longer median OS (18 months *vs*. 14.6 months) and PFS (9.4 months *vs*. 7 months) compared with historical studies using chemoradiotherapy alone. Furthermore, their subsequent phase II clinical study further confirmed the prolonged OS (19.6 months *vs*. 14.6 months) in glioblastoma patients receiving the combination therapy. Additionally, their study found that T2∗ mapping appeared to provide a novel non-invasive, iron-dependent biomarker that can predict the outcomes in glioblastoma patients,^121^ suggesting that tumor iron content may play an important role in the therapeutic effect of ascorbic acid. Furqan et al^122^ found that in metastatic non-small cell lung cancer patients, HDVC combined with standard chemotherapy (carboplatin plus paclitaxel) significantly improved tumor response rates (37.1%–58.9% *vs*. 15%–25%), and prolonged OS and PFS compared with historical controls. Interestingly, subjects with longer PFS exhibited a multi-fold increase in circulating activated CD8 T cells. Although the study did not investigate the potential mechanisms behind this finding, it suggests that HDVC may have systemic immunomodulatory effects. In a randomized trial conducted by Ma et al^30^ in ovarian cancer patients, standard chemotherapy (carboplatin plus paclitaxel) combined with HDVC not only reduced chemotherapy toxicity (*e.g.*, decreases in neurotoxicity, bone marrow toxicity, infection, hepatobiliary toxicity, and renal toxicity) but also significantly prolonged median time to disease progression. Similarly, a randomized trial initiated by Kellie et al^111^ in advanced pancreatic cancer patients found that compared with the standard treatment regimen (gemcitabine plus paclitaxel) group, the combination treatment group had significantly longer OS (16 months *vs*. 8.3 months) and PFS (6.2 months *vs*. 3.9 months). Importantly, no increase in HDVC-related toxicity was observed, and the quality of life was comparable between groups. However, a phase II randomized trial comparing docetaxel versus docetaxel plus HDVC in patients with metastatic castration-resistant prostate cancer was terminated after an interim analysis indicating the ineffectiveness of the primary endpoints. It should be noted, however, that the enrolled patients had previously undergone three or more lines of treatment without any responses. Such extremely late-stage cancer patients are typically insensitive to various treatments and have a poor prognosis.

The above studies suggest that HDVC combined with traditional chemoradiotherapy is well-tolerated, has the potential to reduce toxicity, and can improve treatment efficacy. Large-scale, rigorous randomized controlled trials (RCTs) should be conducted to confirm its efficacy as a first-line supplementary treatment to standard chemoradiotherapy, rather than merely serving as a palliative treatment for extremely late-stage cancer patients.

### Combination with immunotherapy

The preclinical findings described above demonstrate that HDVC plays a crucial role in regulating anti-tumor immune responses, and Furqan et al^122^ also found this potential immunomodulatory effect in a clinical setting. Therefore, combining HDVC with immunotherapy could be a promising approach to enhance the efficacy of immunotherapy.

Currently, clinical studies investigating a combination of HDVC and immunotherapy are scarce. However, a recent case report was the first to describe the clinical application of HDVC in conjunction with immunotherapy.^123^ In this report, two patients with metastatic leiomyosarcoma received treatment with pembrolizumab (200 mg every 3 weeks) combined with weekly 25 g HDVC after radiotherapy. Imaging examinations every 3 months revealed a continuous decrease in tumor burden, showing partial remission. This trend of remission persisted for 12 months after the initiation of treatment. One of these patients initially received pembrolizumab (200 mg every 3 weeks) monotherapy following radiotherapy, but the tumor exhibited poor responses. Subsequently, intravenous vitamin C (25 g weekly) was added to the treatment regimen. Imaging conducted 3 months after combination therapy showed stable disease, and after another 3 months, a CT scan revealed partial tumor remission, which persisted for 17 months after the initiation of the combination therapy. Additionally, HDVC was well-tolerated in all patients and did not produce any additional toxic effects. Given that sarcomas are typically characterized as “cold” tumors with low immune infiltration and limited responses to immunotherapy, this study suggests that HDVC may improve the efficacy of immunotherapy by serving as an immunotherapy sensitizer for “cold” tumors. This finding highlights a potential role of HDVC in enhancing immune responses and warrants further exploration in larger-scale clinical trials.

### Combination with standard therapy

Wang et al^124^ conducted a phase I clinical study to evaluate the efficacy and safety of HDVC combined with chemotherapy and targeted therapy in patients with metastatic colorectal cancer and metastatic gastric cancer. The study consisted of two parts: the first phase explored the safe dosage of HDVC, while the second phase administered a fixed dose of HDVC (1.5 g/kg daily for 3 consecutive days) combined with mFOLFOX6 or FOLFIRI with or without bevacizumab (a vascular endothelial growth factor-targeted drug) in 14-day cycles. The treatment continued for up to 12 cycles. In addition to a good safety profile, the combination therapy also demonstrated potential therapeutic effects (objective response rate of 58.3% and disease control rate of 95.8%). Due to these promising phase I results, the study progressed to a phase III trial, which was published in 2022 (VITALITY Study, NCT04516681).^125^ This study was the first published randomized, open-label, multicenter, phase III study of HDVC combined with standard treatment, enrolling 442 patients who were randomly assigned 1:1 to the control group (FOLFOX ± bevacizumab) or the HDVC group (1.5 g/kg/d, intravenously for 3 h from day 1 to day 3) plus FOLFOX ± bevacizumab. In terms of treatment efficacy, the study observed a numerical trend favoring the HDVC group; however, there were no statistically significant differences in PFS and OS between the two groups.

Interestingly, subsequent subgroup analyses showed that metastatic colorectal cancer patients with RAS mutations had significantly improved PFS (9.2 months *vs*. 7.8 months), and multivariate analysis further indicated that the combination of HDVC was an independent factor for prolonging PFS in patients with RAS mutations. A similar trend was also observed in the analysis of OS (20.2 months *vs*. 16.8 months). This finding aligns with previous preclinical studies demonstrating that high levels of vitamin C have selective cytotoxicity against RAS-mutant colorectal cancer cells.^47^ This outcome suggests that future research and clinical applications of HDVC should consider selecting patients based on RAS mutation status to optimize treatment efficacy. By identifying genetic subgroups likely to benefit from HDVC, personalized treatment approaches may maximize therapeutic outcomes.

### Combination with non-pharmaceutical therapy

Ou et al^126^ conducted a phase I clinical study to evaluate the safety and pharmacokinetics of HDVC in combination with modulated electrohyperthermia in stage III-IV non-small cell lung cancer patients. The enrolled patients had progressed after radiotherapy and/or chemotherapy or were unresponsive to other conventional treatments. The study revealed that fasting plasma vitamin C levels in these patients were significantly lower compared with healthy individuals, and the concentration levels were negatively correlated with advanced disease stages. A combination of HDVC (1.5 g/kg, 3 times per week, injected every other day, for 4 consecutive weeks) and modulated electrohyperthermia showed good tolerability and safety. Moreover, patients showed significant improvements in functional scores and quality of life. Additionally, the study discovered that HDVC could reach the highest plasma concentration when HDVC was concomitantly applied with modulated electrohyperthermia, suggesting that future clinical trials using HDVC as a combination therapy should give special consideration to the administration sequence.

In a subsequent randomized phase II trial,^127^ researchers compared a combination of HDVC plus modulated electrohyperthermia with best supportive care to best supportive care alone in pretreated, advanced, refractory non-small cell lung cancer patients. The results showed that the combination therapy was well-tolerated, with no additional toxic effects observed. Importantly, the combination therapy significantly improved patients' quality of life and prolonged both PFS (3.0 months *vs*. 1.85 months) and OS (9.4 months *vs*. 5.6 months).

These studies highlight a promising avenue for further exploration, particularly in integrating HDVC with non-pharmaceutical approaches to improve treatment outcomes.

### Perspectives on clinical trial design

Overall, most clinical trials conducted to date have enrolled a small number of patients, and large-scale, high-quality RCTs are needed to further evaluate the anti-tumor effects of HDVC. The premise of such research designs is to find the optimal dosage, administration sequence, and administration method. Several studies mentioned above have evaluated the safe dosage of HDVC by gradually increasing the dose. In general, intravenous vitamin C administration of 70–100 g or >1 g/kg, 3–4 times per week, can achieve pharmacological plasma concentrations of vitamin C (≥20 mM) while being well-tolerated by patients.^30^^,^^114^^,^^115^^,^^119^^,^^124^ However, in these studies, patients are far from reaching the maximum tolerated dose, and the selection of the highest injection dose was often based on the plasma concentration reaching a plateau.

**Figure 3:**
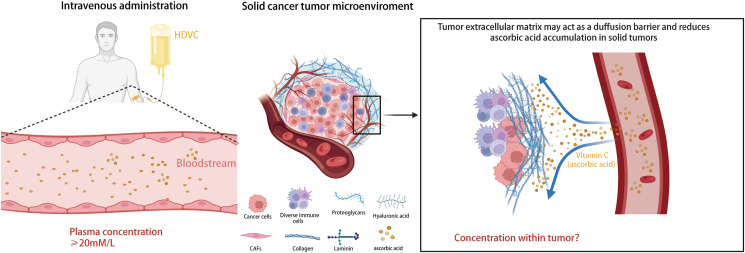
Potential mechanisms underlying the difference between intra-tumoral concentration and plasma concentration of vitamin C.

For non-solid tumors such as multiple myeloma, pharmacological plasma concentrations of vitamin C may be sufficient for therapeutic requirements. However, for solid tumors, it is questionable whether the plasma vitamin C concentration accurately reflects the intra-tumoral concentration. In other words, it is not certain whether solid tumors form a barrier similar to the “immune-excluded microenvironment” affecting the entry of vitamin C into the tumor interior (Fig. 3).

Chen et al^128^ found that in patients with advanced solid tumors, intravenous vitamin C still exhibited first-order kinetics within the dosage range of 75–100 g, but the excretion rate in tumor patients was slightly lower than that in healthy individuals, indicating the utilization of vitamin C in tumors. Dachs et al^42^ found that at physiological concentrations of plasma vitamin C, the level of ascorbic acid in tumors was significantly lower than that in peripheral tissues. When pharmacological concentrations were reached, the levels of ascorbic acid in tumors and normal tissues increased significantly, with a more pronounced increase in tumors compared with normal tissues, accompanied by a decrease in the level of HIF markers within the tumor. These findings suggest that once the plasma vitamin C concentration exceeds a certain threshold, the compound can rapidly penetrate solid tumors, be metabolized, and improve hypoxic TME.

The current research may be too conservative in selecting the dosage. Stephenson et al^27^ showed that intravenous administration of vitamin C at 110 g/m^2^ (approximately 190 g), 4 days/week for 4 consecutive weeks, was well-tolerated. Further studies are needed to investigate the relationship between intravenous dosage and intra-tumoral accumulation in solid tumors. This research could help explain the inconsistent outcomes of current clinical studies, establishing a threshold for effective drug exposure and avoiding unnecessary prolonged infusions in patients.

The study by Ou et al^126^ indicated that concomitant administration with modulated electrohyperthermia could achieve higher plasma vitamin C concentrations, suggesting that future studies should consider pharmacokinetic differences when combining intravenous vitamin C with other treatment modalities (such as chemotherapy) or the tendency of vitamin C to accumulate in specific locations (such as radiotherapy) to achieve optimal synergistic effects. Riordan's dosage study found that intravenous administration of 60 g could achieve a plasma ascorbic acid concentration of 24 mM, but the duration was short. A second intravenous administration of 20 g within 1 h after the first infusion could maintain a plasma concentration of ≥24 mM for 4 h,^129^ highlighting the importance of the infusion regimen.

Future research designs should determine the optimal dosage, sequence, and administration method for HDVC. This is a prerequisite for conducting standardized RCTs, which will be crucial for verifying HDVC's efficacy and establishing its role in cancer treatment.

## Emerging trend

### Nano-vitamin C delivery systems

The stability of vitamin C is influenced by various factors, including temperature, pH, and enzymatic oxidation, which contribute to its degradation and reduced activity. This low stability limits the therapeutic application of vitamin C via intravenous injection.^130^ Additionally, as discussed earlier, the therapeutic effect of vitamin C in solid tumors may be related to its intra-tumoral concentration accumulation. Therefore, it is crucial to utilize an effective delivery system to stably transport vitamin C to the target site.

Nanomaterial-based drug delivery systems have demonstrated the ability to enhance the stability, targeting, and pharmacokinetic activity of drugs *in vivo*.^131^ Consequently, nanomaterial-based vitamin C delivery systems, including liposomes, nanoparticles, and polymers,^132^, ^133^, ^134^ have emerged as promising strategies to address these challenges mentioned above.

#### Pre-clinical studies and clinical translation

Several preclinical studies have demonstrated the tumor-suppressive effects of nanomaterial-based vitamin C. Ma et al constructed vitamin lipid-polymer nanoparticles that can specifically release vitamin C at tumor sites. Both *in vitro* and *in vivo* studies found that such nanoparticles could repolarize M2-type macrophages into anti-tumor M1-type macrophages and promote the infiltration of cytotoxic T cells in the TME. Moreover, when combined with PD-L1 therapy, vitamin lipid-polymer nanoparticles exhibited a stronger immune response.^135^ Gao et al designed a pH-sensitive nanocarrier that can control the release of ascorbic acid in a hypoxic environment, achieving targeted delivery and reversible pH-controlled release of ascorbic acid. *In vitro* and *in vivo* experiments proved that the nanocarrier could target tumor sites and release vitamin C, inducing reductive stress-induced apoptosis in liver cancer cells and inhibiting tumor growth.^136^ Amiri et al designed a liposomal nanocarrier and demonstrated through a series of *in vitro* experiments that compared with free drugs, the nanocarrier-loaded drug significantly enhanced apoptosis and cytotoxicity in MCF-7 breast cancer cells, inhibited cell migration, up-regulated p53 and Bax genes, and down-regulated the anti-apoptotic gene Bcl-2. Furthermore, histopathological examinations showed that breast cancer cells treated with the nanocarrier-loaded drug had lower mitotic index, invasiveness, and pleomorphism.^137^

While preclinical studies highlight the versatility of nanomaterials, including liposomal, polymeric nanoparticles, and lipid polymer hybrid nanoparticles, clinical translation remains predominantly focused on liposomal delivery systems. Regarding the bioavailability of liposomal formulations, several studies have shown that compared with traditional methods, oral nano-vitamin C can significantly improve vitamin C absorption and clearance rates and alter its pharmacokinetics *in vivo*.^138^, ^139^, ^140^ Hickey et al^141^ found that oral liposomal vitamin C can increase circulating vitamin C levels beyond pharmacological concentrations (up to 400 mM/L), and pharmacokinetic analysis showed that repeated administration can maintain high plasma concentrations. Davis et al^142^ showed that the circulating concentrations generated by orally administered liposome-encapsulated vitamin C were higher than those of unencapsulated vitamin C, indicating the higher bioavailability of nano-vitamin C. Although the circulating concentrations were lower than those achieved by intravenous administration, the protective effects against ischemia-reperfusion-mediated oxidative stress were similar. These results suggest that while blood is the main transport medium for vitamin C between the intestine and other tissues, the plasma concentration of vitamin C may have a therapeutic “threshold”. Once the circulating concentration reaches this “threshold”, the therapeutic effect of vitamin C may be more related to its concentration in tissues/cells. Furthermore, Purpura et al^143^ found that oral liposomal vitamin C not only increased plasma concentrations of vitamin C but also significantly elevated its concentration in leukocytes. Given that the binding of vitamin C to leukocytes is associated with its immunomodulatory effects, this study suggests that nano-vitamin C more readily enters cells and may exert stronger immunomodulatory functions. These studies further indicate that the accumulation of vitamin C in tissues/cells may be associated with better therapeutic outcomes.

Currently, clinical trials on nano-vitamin C remain scarce, and available clinical investigations have been predominantly conducted in non-cancer populations. A study on upper respiratory tract infections suggested that oral liposomal vitamin C could improve children's immunity and alleviate symptoms of upper respiratory tract infections, serving as an adjuvant treatment for children with upper respiratory tract infections.^144^ A study conducted during the COVID-19 pandemic reported that oral high-dose liposomal vitamin C could reduce the mortality rate of COVID-19 patients. Among the 8634 patients involved in the study, the mortality rate in the experimental group (nano-vitamin C) was 1.9%, while the mortality rate in the control group (untreated) was 4%. Additionally, a study conducted in patients with persistent symptoms/signs after acute COVID-19 (long COVID) showed that long COVID patients who received 500 mg of oral liposomal vitamin C had significantly improved mobility, muscle strength, endothelial function, and fatigue symptoms compared with the placebo group.^145^^,^^146^ A study conducted in patients with skin melasma demonstrated that when the ascorbic acid derivative magnesium ascorbyl phosphate was formulated as a liposome, it significantly enhanced its skin penetration and retention while maintaining sustained stability.^147^ This suggests that the high penetration and retention of nano-vitamin C in tissues is worth further promoting for therapeutic applications in solid tumors.

#### Nano-vitamin C versus intravenous infusion

**Figure 4:**
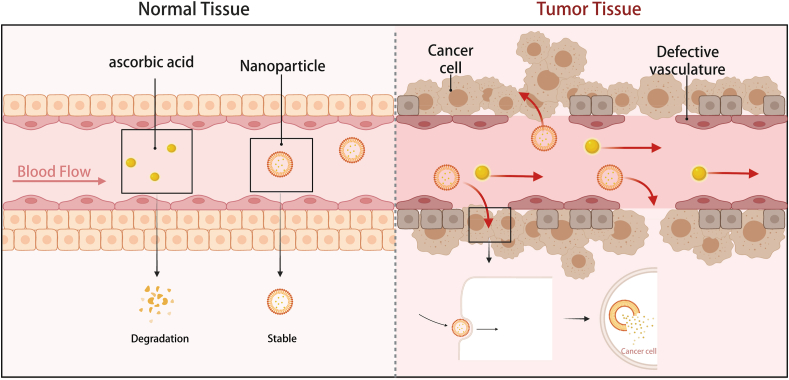
The potential advantages of nano-vitamin C: i) greater stability during transport in the complex blood system; ii) improving the cellular uptake of vitamin C; and iii) achieving targeted release.

Compared with traditional molecular form through intravenous infusion, nano-vitamin C has three specific advantages (Fig. 4) in cancer treatment: i) Encapsulation or chemical conjugation provides greater stability for vitamin C during transport in the complex bloodstream. Studies have shown that nanoparticle-formulated vitamin C only undergoes about 5% oxidative degradation after 10 days of storage at room temperature.^148^ ii) Cellular uptake is enhanced. Molecular vitamin C is oxidized to dehydroascorbic acid, which enters cells through Na^+^-dependent and energy-dependent transporters and is then regenerated into vitamin C for biochemical metabolism. In contrast, nano-vitamin C is mainly taken up by cells through endocytosis or phagocytosis (nanoparticles smaller than 200 nm are mainly taken up by cells through clathrin-mediated endocytosis, while nanoparticles larger than 500 nm enter cells through phagocytosis), greatly improving the cellular uptake of vitamin C.^149^^,^^150^ iii) The release is better controlled and targeted. By modifying the composition of nanoparticles, the controlled release of vitamin C in different environments can be achieved. For example, carrier designs targeting abnormal blood vessels, hypoxia, and acidic pH can enable targeted release of vitamin C in the TME.^151^ In addition, compared with intravenous infusion, nano-vitamin C offers targeted release capabilities, enhanced cellular uptake efficiency, and superior stability for vitamin C. Critically, due to rapid renal excretion, vitamin C struggles to sustain pharmacological concentrations in plasma over prolonged periods. Intravenous infusion, as an invasive procedure, poses practical limitations for sustaining therapeutic levels through repeated injections. In contrast, nano-vitamin C, delivered orally, achieves pharmacological concentrations while enabling sustained maintenance through non-invasive dosing. Furthermore, nano-vitamin C exhibits superior tissue/cellular bioavailability, rather than transient plasma levels, which may translate to enhanced therapeutic efficacy.

#### Perspective on nano-vitamin C

It is crucial to emphasize that the current clinical studies have been conducted exclusively in non-oncological contexts, with no cancer-specific clinical trials reported to date. However, existing studies mentioned above suggest that oral nano-vitamin C can achieve satisfactory plasma concentrations, exhibit high tissue and cellular bioavailability, and provide potential immunomodulatory effects, providing a compelling rationale for exploring this modality in cancer therapy and translating these insights into clinical applications. Future preclinical studies should focus on preparing more precise targeting carriers and investigating the accumulation of vitamin C in tissues/cells. More clinical studies on the effects of nano-vitamin C in solid tumor patients should be conducted to provide concrete evidence for its therapeutic efficacy and benefit patient populations.

### Screening of patients sensitive to HDVC

Currently, the mechanism of HDVC as an adjuvant for anti-tumor treatment has not been fully elucidated, and the lack of standardized clinical trial designs has led to inconsistent conclusions across studies, resulting in a disconnect between clinical and preclinical research findings. This gap hinders the development of large-scale RCTs, preventing researchers from using the “gold standard” to verify HDVC's efficacy and creating a vicious cycle. In addition to efforts to standardize dosage and administration routes, screening patients sensitive to HDVC treatment is crucial for the design of future clinical trials.

Tian et al^70^ found that the Warburg effect induced by HIF pathway activation greatly enhances the toxicity of vitamin C in multiple tumor cell lines. Elevated HIF increases the cellular uptake of vitamin C by targeting GLUT1, thereby inducing metabolic exhaustion within cancer cells. This finding suggests that tumor cells with high HIF expression may represent a potential metabolic phenotype sensitive to HDVC treatment.

A recent article^152^ provided a systematic review of vitamin C intracellular transporters in tumors. In normal cells, vitamin C uptake mainly relies on two types of transporters, GLUT and SVCT. However, SVCT is rarely expressed on the plasma membrane of tumor cells that primarily take up vitamin C in its oxidized form. Therefore, tumor cells with high GLUT1 expression have a stronger ability to take up vitamin C and may be more sensitive to HDVC treatment.

In addition to metabolic phenotypes, mismatch repair gene mutations may also be potential therapeutic targets for HDVC.^153^ Myeloid neoplasia, characterized by epigenomic and genomic abnormalities, frequently exhibits TET2 mutations. Guan et al^154^ demonstrated that ascorbic acid could reduce the proliferation of TET2-mutant myeloid neoplasia by promoting iron redox reactions and restoring dioxygenase activity. TET2 mutations and isocitrate dehydrogenase (IDH) mutations are recurrent and mutually exclusive in the acute myeloid leukemia genome. Mingay et al^155^ found that HDVC treatment could induce decreased cell proliferation and increased expression of leukocyte differentiation genes in a leukemia model expressing IDH1R132H. A case report^156^ described the therapeutic effect of vitamin C in a patient with acute myeloid leukemia (with THE2 and WT1 mutations) who did not respond to induction chemotherapy. Subsequent HDVC treatment induced a sustained clinical remission in the patient for two years. These studies suggest that HDVC can induce epigenetic remodeling in myeloid neoplasia and may serve as an adjuvant therapy for patients with mutations in the IDH1/2-TET2-WT1 pathway. They also suggest that there is a lack of appropriately gene-stratified patient cohort studies to demonstrate the anti-tumor benefits of vitamin C.

For patients with colorectal cancer, the aforementioned RCT by Wang et al^125^ found that colorectal cancer patients with KRAS/BRAF gene mutations were more likely to benefit from HDVC treatment. Additionally, Shi et al performed an analysis of dietary and tumor molecular data from 2096 patients enrolled in two U.S.-based prospective cohorts.^157^ Their findings revealed that a higher total vitamin C intake was significantly associated with reduced colorectal cancer-specific mortality in patients with KRAS or BRAF mutations, compared with those with wild-type tumors. These findings support the potential role of vitamin C as an adjuvant therapy for colorectal cancer patients with KRAS/BRAF mutations.

The preclinical and clinical studies mentioned above suggest that as our understanding of the anti-tumor mechanisms of HDVC continues to evolve, future clinical research should focus on stratifying patients based on metabolic and genetic phenotypes. Such an approach would enable precision treatment with HDVC and provide robust evidence for its therapeutic efficacy in well-defined patient populations.

## Conclusion and future perspective

More than half a century has passed since the anti-cancer effects of vitamin C were first proposed. During this time, it has experienced a tidal wave of emergence, decline, and resurgence. With deeper exploration of its mechanisms, vitamin C shows great promise as an economical and non-toxic anti-tumor agent. Today, an increasing number of well-designed, high-impact preclinical and phase I/II clinical trials are driving the vigorous development of this field. To make HDVC more widely applicable to various cancer patients, large-scale phase III RCTs are urgently needed to test its efficacy in combination with standard treatment regimens.

Regarding side effects, current concerns mainly focus on the potential for HDVC to cause kidney stone formation and the possibility of hemolytic anemia in patients with glucose-6-phosphate dehydrogenase (G6PD) deficiency after vitamin C infusion.^25^ To ensure participant safety, renal function and G6PD status should be carefully assessed during patient screening.

Regarding the optimal administration regimen, this review indicates that HDVC combination therapy administered intravenously at least 2–3 times per week, at 75–100 g or >1.0 g/kg, for 6–8 cycles, can achieve pharmacological concentrations in the plasma while ensuring safety. In terms of administration sequence, since most current clinical trials combine HDVC with chemotherapy, simultaneous infusion is often used. However, when combining HDVC with site-specific therapies such as radiotherapy, the distribution and accumulation of vitamin C in the circulatory system should be carefully considered to achieve optimal efficacy. Regarding intravenous dosage, current pharmacokinetic studies suggest that the doses used in most clinical studies remain far below the maximum tolerated dose. Considering that higher doses may be associated with higher penetration into and longer maintenance times in the solid tumors, future studies can appropriately increase the infusion dose in the properly screened patients. Additionally, due to factors such as solid tumor hardness, peripheral exclusion, and abnormal neovascularization, it is critical to distinguish between plasma vitamin C concentrations and intra-tumoral concentrations. Future research should focus on measuring intra-tumoral concentrations and metabolic utilization of vitamin C after intravenous injection to determine the optimal dosing and administration strategy.

Another challenge lies in the instability, rapid oxidative metabolism and degradation, and poor bioavailability of vitamin C *in vivo*. Moreover, the long duration of intravenous administration, discomfort, and inability to target solid tumors limit the clinical application of HDVC to a certain extent. Nano-vitamin C offers a promising solution to overcome these limitations. By improving the stability and bioactivity of vitamin C, nanoformulations may address these challenges while enhancing therapeutic efficacy. Although this field is still at its early stage, several preclinical studies have shown the potential of nano-vitamin C to improve tumor targeting and immunomodulation. Future research on nano-vitamin C formulations should focus on elucidating their mechanisms and therapeutic effects. Additionally, clinical trials are needed to provide conclusive evidence for their efficacy and to explore their potential in solid tumor treatment.

Most current studies on HDVC combination therapies focus on standard chemoradiotherapy. In recent years, cancer immunotherapy has shown promising results in treating various tumor types. However, its efficacy in solid tumors remains controversial, as many solid tumors (*e.g.*, colorectal and pancreatic cancers) are classified as “cold tumors” due to low immune cell infiltration and low expression of immune checkpoints, leading to poor treatment outcomes. Preclinical studies on HDVC have found that it has a strong immunomodulatory effect, and the only case report available so far has reported encouraging results from the combined application of HDVC and immunotherapy. Therefore, future HDVC combination therapy clinical trials should consider cancer immunotherapy to demonstrate its immune-sensitizing effect in converting “cold tumors” into “hot tumors".

In conclusion, while clinical trials are essential to confirm HDVC's efficacy, deeper mechanistic studies are equally important to ensure that this resurgent anti-cancer agent is not prematurely dismissed again. With ongoing research and development, ascorbic acid has the potential to become an important component of cancer treatment and prevention strategies. By addressing current challenges and exploring new therapeutic combinations, HDVC may soon play a significant role in practical oncology.

## CRediT authorship contribution statement

**Hanzheng Zhao:** Writing – review & editing, Writing – original draft, Conceptualization. **Wentao Fu:** Writing – review & editing, Writing – original draft, Conceptualization. **Xiaobao Yang:** Visualization, Investigation. **Wenhui Zhang:** Visualization, Investigation. **Si Wu:** Visualization, Investigation. **Jingxin Ma:** Visualization, Validation, Investigation. **Tianzhen Zhang:** Writing – review & editing, Supervision, Funding acquisition, Conceptualization. **Hongwei Yao:** Writing – review & editing, Supervision, Funding acquisition, Conceptualization. **Zhongtao Zhang:** Writing – review & editing, Supervision, Funding acquisition, Conceptualization.

## Funding

This work was supported by the National Key Technologies R&D Program of China (No. 2017YFC0110904), Noncommunicable Chronic Diseases-National Science and Technology Major Project (China) (No. 2024ZD0520302), special funding support of Beijing Hospitals Authority Clinical Medicine Development (China) (No. ZLRK202302), 10.13039/501100001809National Natural Science Foundation of China (No. 82101923), and Beijing Municipal Administration of Hospitals' Youth Programme (China) (No. QML20230106).

## Conflict of interests

The authors declared no conflict of interests.
